# The Avian Proghrelin System

**DOI:** 10.1155/2010/749401

**Published:** 2010-02-10

**Authors:** Mark P. Richards, John P. McMurtry

**Affiliations:** United States Department of Agriculture, Agricultural Research Service, Animal Biosciences and Biotechnology Laboratory, Beltsville Agricultural Research Center, Animal and Natural Resources Institute, Beltsville, MD 20705-2350, USA

## Abstract

To understand how the proghrelin system functions in regulating growth hormone release and food intake as well as defining its pleiotropic roles in such diverse physiological processes as energy homeostasis, gastrointestinal tract function and reproduction require detailed knowledge of the structure and function of the components that comprise this system. These include the preproghrelin gene that encodes the proghrelin precursor protein from which two peptide hormones, ghrelin and obestatin, are derived and the cognate receptors that bind proghrelin-derived peptides to mediate their physiological actions in different tissues. Also key to the functioning of this system is the posttranslational processing of the proghrelin precursor protein and the individual peptides derived from it. While this system has been intensively studied in a variety of animal species and humans over the last decade, there has been considerably less investigation of the avian proghrelin system which exhibits some unique differences compared to mammals. This review summarizes what is currently known about the proghrelin system in birds and offers new insights into the nature and function of this important endocrine system. Such information facilitates cross-species comparisons and contributes to our understanding of the evolution of the proghrelin system.

## 1. Introduction

Mammalian ghrelin is a 28 amino acid peptide hormone with a unique fatty acylation (n-octanoyl) modification at the serine 3 (Ser^3^) position that exhibits growth hormone (GH) releasing and orexigenic activities. It was originally isolated from rat stomach and shown to be the endogenous ligand for the growth hormone secretagogue receptor (GHS-R) in rats and humans [1]. The first report of the existence of avian ghrelin came in 2002 with the purification of the peptide, cloning of a cDNA, and preliminary testing of biological activity of the ghrelin peptide isolated from the proventriculus (the glandular portion of the avian stomach) of chickens [2]. Since that initial report, preproghrelin genes and predicted amino acid sequences for six different avian species have now been identified [3–5]. There have also been a number of reports concerning the structure and expression of the GHS-R gene in chickens, quail, and ducks. While some functions such as GH release are conserved in birds, other actions such as the effect of ghrelin on food intake are opposite to what has been found in mammals and other vertebrate species [3–5]. Moreover, the majority of studies in birds have focused on the ghrelin/GHS-R axis almost exclusively. Therefore, it is of interest to study the avian proghrelin system to determine the reasons for such differences and how they might relate to the evolution of this important hormonal system. This review focuses on the major components of the avian proghrelin system and discusses what is currently known about their structure, expression, and physiological action, while highlighting potential areas of future investigation needed to better define the operation of this system in birds.

## 2. Structure and Expression of the Preproghrelin Gene

To date, preproghrelin genes have been sequenced and characterized for the chicken, turkey, and duck (GenBank accession nos. AY303688, AY497549, and EF613552, resp.). In addition, cDNA sequence is now available for goose, quail, and emu preproghrelin mRNA (GenBank accession nos. AY338465, AB244056, and AY338467, resp.). Based on this evidence it has been possible to project some of the features and genomic organization of a prototypical avian preproghrelin gene, as well as, to compare and contrast sequence variations that may be associated with functional traits in different avian species.

### 2.1. Gene Structure and Genomic Organization

**Figure 1:**
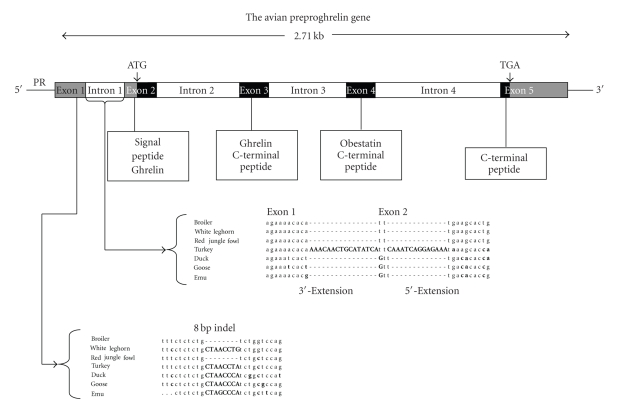
Genomic structure and organization of a prototypical avian preproghrelin gene (based on the chicken gene). The gene exhibits five exons (four coding exons shown in black) and four introns with the positions of the proximal promoter (PR) and the start (ATG) and stop (TGA) codons indicated. The portions of the preproghrelin precursor protein encoded by each exon are indicated in boxes. Also indicated are two sequence features detected in different avian genes. These include an 8 bp insertion/deletion (INDEL) located in exon 1 and exon extensions (5′ and 3′) of exons 1 and 2 detected only in the turkey.

The avian preproghrelin gene consists of five exons and four introns (Figure 1). This structure is similar to the human gene with the exception of the first exon which is larger in birds [6]. The transcriptional unit for the chicken preproghrelin gene locus, located on chromosome 12, spans 2.71 kb [7, 8]. The preproghrelin protein precursor is encoded within exons 2–5, with the mature ghrelin peptide split between exons 2 and 3 and obestatin encoded entirely within the fourth exon (Figure 1). 

A recent study, that reexamined the structure and genomic organization of human and mouse preproghrelin genes, reported the existence of novel exons (including a sixth exon) containing 5′-untranslated region (5′-UTR) sequence and potential new open reading frames upstream of the originally identified first exon [9]. The existence of novel exons was confirmed by examining gene transcripts in cDNA libraries from different tissues using rapid amplification of cDNA ends (5′ RACE). No additional exons or alternatively spliced transcript variants have been reported for any avian preproghrelin gene to date. In light of this, it is interesting to note that evidence could not be found for conserved regions containing the novel upstream exons in chicken genome sequence which suggests that these novel exons may have evolved subsequent to the divergence of birds and mammals [9]. However, these findings do not rule out the possibility that a more complex structure and organization for the preproghrelin gene locus may exist in other species including birds. This is an area that clearly merits further investigation.

### 2.2. Sequence Variations

Analysis of different avian preproghrelin gene sequences revealed the existence of a number of polymorphisms, some of which could have functional significance. One particularly interesting feature is an 8 bp indel (insertion/deletion) found in the first exon (a noncoding exon) of egg-layer chickens (White Leghorn) that was absent in meat-type (broiler) chickens (Figure 1). This may be an important finding because these two strains differ markedly in their appetite and growth characteristics. The indel was also absent in red jungle fowl, an ancestral species for modern commercial chicken breeds [8]. This unique feature has been reported to occur at low frequency in a study summarizing sequence polymorphisms in four chicken breeds [7]. Moreover, sequence similar (but not identical) to the chicken indel and of the same size (8 bp) was present in the same location (exon 1) in the turkey, goose, duck, and emu [8, 10]. The presence of the 8 bp indel has subsequently been found to be positively associated with growth and carcass traits in meat-type chickens [11]. The turkey ghrelin gene also contains additional sequence (30 bp) at the junction of exons 1 and 2 due to exon extension (Figure 1). A single-base extension of exon 2 was found in duck, goose, and emu sequences [10]. Since both of these sequence features occur outside of the coding region of the mRNA transcript, their effects on preproghrelin gene function, if any, remain to be determined. It is possible that they could influence posttranscriptional processes such as mRNA stability or translation efficiency.

A single-nucleotide polymorphism (SNP, C223G) occurring in the 5′-UTR of the chicken preproghrelin gene was reported to determine the presence or absence of a specific transcription factor binding site (serum response factor) that could potentially influence expression of the ghrelin gene [7]. This group also reported a total of 19 SNPs in the chicken ghrelin gene sequence, the majority of these occurring within the 4 introns. There were no significant differences in these polymorphisms between egg-layer and meat-type chickens [7]. Nie et al. [7] and Richards et al. [8] reported an SNP (A/G) found in exon 5 (within the coding region) of the chicken preproghrelin gene that results in an amino acid change (Gln113Arg). However, this change would affect the C-terminal peptide region of the prohormone precursor and not in the mature ghrelin or obestatin peptides. Thus, its functional impact is unknown. There have been reports of an amino acid sequence variant form of ghrelin (des-Gln^14^) in rats and humans that is created by the use of an alternative splice donor site located at the intron 2/exon 3 boundary. This change could potentially affect the physiological actions of mature ghrelin. However, when the purified peptide was tested, its activity (i.e., receptor binding) was comparable to intact ghrelin [12]. Moreover, this variant form is not produced at the same levels in stomach or plasma as intact ghrelin [13]. Sequence reported for all avian preproghrelin genes does not provide any evidence for an alternative splicing event that could lead to the same type of amino acid deletion in the mature avian ghrelin peptide.

### 2.3. Promoter Region

Flanking upstream (5′) sequence (2 kb) was examined in an attempt to determine characteristics of the putative proximal promoter region and perhaps shed some light on factors that might regulate preproghrelin gene expression in chickens [8]. Little is known about transcriptional regulation of the human gene [6, 9, 14–16] and nothing is currently known about preproghrelin gene regulation in birds. Glucagon and its second messenger, cAMP, have been reported to enhance human preproghrelin gene transcription and this has been suggested as a possible mechanism that leads to increased plasma ghrelin levels in response to fasting [15]. Similarly, growth hormone releasing hormone (GHRH) was found to upregulate gene transcription in the pituitary via a cAMP-signaling cascade [16]. The identification of two cAMP response element binding protein (CREB) sites in the putative promoter region of the chicken preproghrelin gene is consistent with the proposed gene regulation mechanism involving cAMP [8]. While an SREBP-1 site was identified just upstream of the TATA box in the chicken gene [8], a role for this transcription factor in preproghrelin gene regulation has not been reported previously in any species. The presence of multiple SRY and SOX-5 sites was also noted in this region of the chicken gene. However, the significance of these findings to preproghrelin gene regulation remains to be demonstrated. 

Recent evidence suggests the involvement of more than one promoter (proximal and distal) in regulating transcription of the human preproghrelin gene to generate transcripts with differing 5′-ends and coding regions [6, 9]. There have been no reports of a corresponding alternate (distal) promoter in any avian preproghrelin gene to date. However, such findings emphasize the need for reexamination of the preproghrelin gene promoter(s) in different species to determine if such diversity exists and how that might relate to gene regulation.

### 2.4. mRNA Expression

Kaiya et al. [2] found preproghrelin mRNA expressed in proventriculus, brain, lung, spleen, and intestine among various chicken tissues surveyed. We reported expression of this mRNA in all tissues examined from 3-week-old male broiler chickens [8]. Proventriculus showed the highest expression followed by pancreas, brain, and intestine. In general, all studies to date indicate that the proventriculus is the predominant site for preproghrelin mRNA expression in birds reflecting an important systemic or endocrine role for the proventriculus with respect to regulating blood ghrelin levels [3–5]. However, the expression of preproghrelin mRNA in a variety of central and peripheral tissue sites also indicates the potential for local (autocrine/paracrine) effects of the proghrelin system in birds. Saito et al. [17] quantified preproghrelin mRNA levels in different regions of the brain in chickens and found that the corpus striatum expressed the highest levels followed by the cerebellum, the optic lobes, and the brainstem.

A number of studies have demonstrated the influence of feeding state on preproghrelin mRNA expression in different avian tissues [3–5]. Fasting has been reported to increase preproghrelin mRNA levels in proventriculus, but refeeding following a period of fasting did not return these levels to prefasting values [8, 18, 19]. The up-regulation of mRNA levels in the proventriculus during fasting coincides with changes in plasma acylated-ghrelin indicating an important role for the proventriculus in determining circulating hormone levels [18]. Effects of fasting on other tissues expressing preproghrelin mRNA included a reduction in pancreas, no effect on whole brain or hypothalamus, and an increase in liver [8, 18, 19]. Since the levels of mRNA expression are much lower in these tissues compared to the proventriculus, these effects most likely reflect localized (autocrine/paracrine) actions of the proghrelin system.

**Figure 2:**
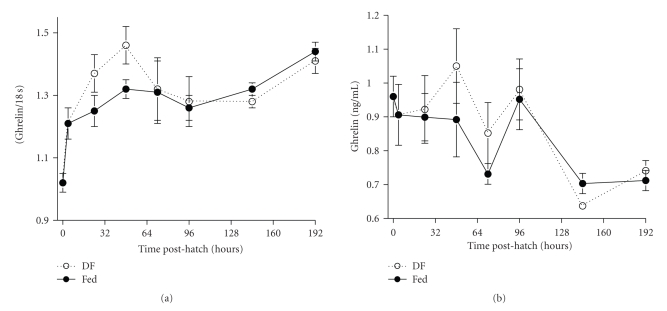
Expression of preproghrelin mRNA (a) in proventriculus and plasma total (acylated + des-acyl) ghrelin levels (b) in two groups of neonatal broiler chicks from hatch to 8 days posthatch. Feed was provided immediately after hatching (Fed) or was withheld for the first 48 hours after hatch (delayed feeding, DF). Values represent the mean ± SEM (*n* = 6).

There have been a few studies detailing developmental changes in preproghrelin mRNA expression during embryogenesis and during the early post hatching period. Wada et al. [20] observed preproghrelin mRNA expression only in the proventriculus of newly hatched White Leghorn chicks, whereas in adult birds, mRNA expression was also detected in duodenum. They also found that mRNA and protein expressions were similar in proventricular mucosal X/A-like endocrine cells from adult chickens, whereas in newly hatched chicks, there was higher mRNA as compared to protein expression. Chen et al. [19] found low expression of preproghrelin mRNA in the proventriculus between days 15 to 19 of embryonic development, but expression increased dramatically by 2 days posthatch. We also found a similar ontogenic pattern from hatch (day 0) to 8 days posthatch (Figure 2(a)), with a peak at 2 days and a delay (48 hours) in feeding further enhanced the up-regulation. Furthermore, plasma levels of total ghrelin followed mRNA expression up to 2 days posthatch and then diverged with plasma ghrelin declining by 8 days posthatch (Figure 2(b)). The latter finding reflects the observations of Wada et al. [20] and Yamato et al. [21] reporting differences in mRNA expression and ghrelin protein levels in the proventriculus of newly hatched chicks and adult birds. The divergence in circulating ghrelin levels and mRNA expression in proventriculus could depend on events related to the transition from embryo to hatched chick. The perinatal period in birds is characterized by a major metabolic shift from the utilization of a high fat nutrient source (yolk) to a high carbohydrate diet (feed). During the first few days posthatch, the newly emerged chick begins feeding and ceases to rely solely on residual yolk from the yolk sac which is rapidly absorbed. Immediately after hatching (i.e., within the first 3 days), there is a dramatic up-regulation of preproghrelin mRNA expression in the proventriculus [19–21] and Figure 2(a). It has been suggested that the availability of specific nutrients (especially fatty acids such as octanoic acid) from the diet being consumed could influence the posttranslational processing (acylation) of the proghrelin protein precursor and secretion of the mature ghrelin peptide by proventricular X/A-like cells [19–21]. Thus, the divergent patterns of preproghrelin mRNA expression in the proventriculus and plasma ghrelin levels by four days posthatch may reflect differential maturation of transcriptional and posttranscriptional regulatory processes in the newly hatched chick which depend both on developmental and nutritional factors.

## 3. Proghrelin Precursor-Derived Peptides and Posttranslational Processing

**Figure 3:**
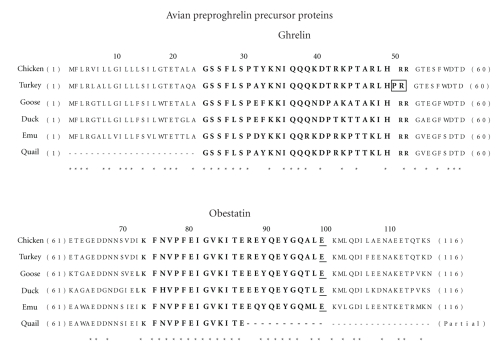
Amino acid comparisons of preproghrelin precursor proteins for different avian species. The locations of the mature ghrelin peptide and the putative obestatin peptide are indicated by larger bold typeface. The C-terminal glutamic acid residue (E) conserved in avian obestatin peptides is shown by bold underline typeface. Also, basic amino acids delineating proteolytic cleavage sites are shown in bold italic typeface. *indicates amino acid identity across all species. Amino acid sequences shown for chicken (broiler), turkey, goose, duck, and quail preproghrelin precursor proteins were obtained from GenBank accession nos. BAC24980, AAP93133, AAQ56122, AAQ56123, and BAE54265, respectively.

**Figure 4:**
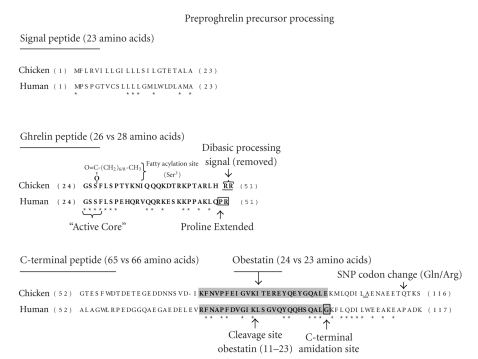
Comparison of amino acid sequence for chicken (GenBank accession no. NP_001001131) and human (GenBank accession no. NP_001128413) perproghrelin precursor proteins and the major peptides processed from the precursor including a signal peptide, the mature ghrelin peptide, C-terminal peptide, and obestatin (shown in gray box). *indicates amino acid identity. The C-terminal glycine (G) residue used to amidate the human obestatin peptide is indicated. Basic amino acids delineating proteolytic cleavage sites are shown in bold italic typeface. The Ser^3^ acylation site within the “active core” of the mature ghrelin peptide is indicated. The location of a single nucleotide polymorphism (SNP) resulting in an amino acid change (glutamine/arginine) located in the C-terminal peptide of the chicken precursor is shown.

An examination of cDNA sequences corresponding to six different avian preproghrelin mRNA transcripts predicts a consensus 116 amino acid preproghrelin precursor protein in birds (Figure 3). Cleavage of the 23 amino acid signal peptide yields a 93 amino acid proghrelin peptide. The proghrelin peptide is then further processed via a series of steps to yield a 26 or 28 amino acid ghrelin peptide and a 65 amino acid C-terminal peptide (C-ghrelin). C-ghrelin contains a putative 24 amino acid obestatin peptide which is generated after further proteolytic processing (Figure 4). The mechanism for producing mature ghrelin peptide (and possibly obestatin) from the mammalian proghrelin precursor was shown to involve limited proteolytic cleavage at a single arginine residue by the prohormone convertase PC1/3 expressed in mouse stomach [22]. A recent report found that PC1/3, PC2, and furin could process proghrelin to yield the mature ghrelin peptide in cultured mammalian cells [23]. We have previously shown that the chicken proventriculus expresses mRNA for both PC1/3 and PC2 and suggested the involvement of these two prohormone convertase enzymes in the processing of proglucagon, another prohormone precursor [24]. However, the precise steps involved in the posttranslational processing of the avian proghrelin precursor have yet to be determined experimentally.

### 3.1. Ghrelin

To date, there has been only one report of the isolation, purification, and characterization of an avian proghrelin-derived peptide, that being ghrelin [2]. The mature acylated ghrelin peptide was isolated from chicken proventriculus tissue based on its ability to bind to and signal through the rat GHS-R in a cell-based assay system [2]. Chicken ghrelin was found to be a 26 amino acid peptide that exhibited 54% of amino acid sequence identity with human ghrelin (Table 1). The first seven amino acids (GSSFLSP) of chicken ghrelin are identical to human ghrelin and it is this N-terminal segment that contains the so-called “active core” (GSSF). The active core is thought to be important in binding to and activation of the GHS-R because it is at the third position (Ser^3^) that attachment of a fatty acid molecule takes place which is known to be essential for ghrelin activation and receptor binding [1–5]. In fact, all avian ghrelin peptides display total conservation of the first seven N-terminal amino acids (Figure 3). Moreover, all avian mature ghrelin peptides are predicted to be 26 amino acids in length with the exception of turkey ghrelin. The turkey mature ghrelin peptide is predicted to be 28 amino acids in length due to a proline-extension at its C-terminal end, similar to human ghrelin (Figures 3 and 4). The other five avian ghrelin peptides contain two arginine residues (RR) at this position which are thought to be cleaved by a carboxypeptidase E-like enzyme during processing to produce the mature 26 amino acid ghrelin peptide [4].

Kaiya et al. [2] determined the acylation state of purified chicken ghrelin peptides and found that the Ser^3^ position was acylated with a medium-chain saturated fatty acid, either octanoic (C8:0) or decanoic (C10:0) acid. Preliminary evidence also suggests the possible acylation of chicken ghrelin with a monounsaturated fatty acid, decenoic acid (C10:1) as has been reported for human ghrelin previously [5, 25]. The mechanism for the acylation and activation of ghrelin has recently been shown to involve the membrane-bound enzyme, ghrelin-O-acyltransferase or GOAT, that attaches medium-chain fatty acids to proteins [26, 27]. Furthermore, it has also been suggested that the posttranslational acylation of the proghrelin precursor occurs independently of the proteolytic processing by PC enzymes so that octanoylation does not require prior cleavage of the mature ghrelin peptide from the proghrelin precursor [22]. The presence of PC enzymes, GOAT, and n-octanoic acid in the culture medium were all found to be required to produce n-octanoyl ghrelin by mammalian cells [23]. 

In mammals, GOAT has been suggested to function as a gastric lipid sensor linking nutrient ingestion (i.e., presence of medium-chain fatty acids and medium-chain triacylglycerols) with actions of the ghrelin endocrine system in maintaining energy homeostasis [28]. There is some evidence to suggest a linkage between gastric lipid sensing and ghrelin activation (GOAT-ghrelin system) in birds. First, analysis of the chicken genome sequence indicated the presence of a GOAT gene ortholog (Mboat4, still uncharacterized) which is located on chromosome 4 [26]. Second, and most intriguing, is the report of Yamato et al. [21] who demonstrated that oral feeding or intraperitoneal (ip) injection of octanoic acid increased the level of octanoylated ghrelin peptide without affecting preproghrelin mRNA levels in neonatal chick proventriculus. These findings strongly suggest that medium-chain fatty acids absorbed from food can be directly utilized to acylate the ghrelin peptide in birds. 

There have been a few reports in which plasma levels of ghrelin were measured in chickens and Japanese quail [8, 18, 29]. The levels detected varied widely however. We determined the plasma levels of total ghrelin in fasted and fed 3-week-old [8] and neonatal (Figure 2) broiler chickens. These levels were substantially higher than those reported by Kayia et al. [18] in fasted and fed layer chicks and Shousha et al. [29] in adult male Japanese quail (ng/mL versus fmol/mL). The reason for these discrepancies involves the different assays employed to measure ghrelin [5]. The commercial assay used to measure total ghrelin (Linco Research, St. Charles, MO) detects both acylated and des-acyl ghrelin, whereas, Kayia et al. [18] and Shousha et al. [29] utilized a specific N-terminal directed radioimmunoassay (RIA) and sample purification (HPLC) to measure only the acylated form of ghrelin. Taken together, the combined results suggest that the level of des-acyl ghrelin is much higher than acylated ghrelin in plasma of birds. However, that has yet to be confirmed by direct measurement of both proghrelin-derived peptides in the same samples. The observations on plasma ghrelin in birds are consistent with mammalian studies which found that des-acyl ghrelin is considerably more abundant in plasma than acylated ghrelin [25, 30]. The significance of this disparity in levels is unclear; however, it is possible that both forms of ghrelin have important physiological roles in birds.

### 3.2. Des-acyl Ghrelin

As indicated above, indirect evidence from plasma ghrelin analyses points to the existence of des-acyl ghrelin in avian blood. The production of des-acyl ghrelin in mammals is thought to occur by deacylation of mature ghrelin occurring both in the gastric mucosa and in plasma under the action of esterase (butrylcholine esterase, paraoxonase, and lysophospholipase I) enzymes [30]. Yang et al. [26] reported that des-acyl ghrelin is not an intermediate of proghrelin processing and suggested that deacylation of mature ghrelin is the mechanism for its production as opposed to its derivation from the proghrelin precursor directly. To date, the existence of ghrelin peptide lacking an acyl-modification of Ser^3^ in avian blood or tissues has yet to be confirmed experimentally [5]. Intracerebroventricular (icv) administration of synthetic des-acyl ghrelin to chickens produced no effect on feeding behavior [3]. Furthermore, in vitro administration of des-acyl ghrelin had no effect on gastrointestinal tract contractility in chickens [31]. Together these results suggest that des-acyl ghrelin does not bind to or activate the GHS-R in birds. 

There have been no studies yet to determine if des-acyl ghrelin can antagonize the actions of ghrelin in birds when coadministered as has been observed in other vertebrate species such as the goldfish [4]. Despite its apparent lack of effect in chickens, growing evidence in mammals suggests that des-acyl ghrelin is active in a number of biological processes in vitro such as stimulating adipogenesis, decreasing hepatocyte glucose output, preventing cell death in cardiomyocytes, and inhibiting cellular proliferation in breast cancer cells [30]. Since it is generally recognized that des-acyl ghrelin does not interact with the GHS-R [1], these actions are thought to be mediated by a different receptor(s) which has yet to be identified [13].

### 3.3. Obestatin

Zhang et al. [32] originally identified obestatin as a proghrelin-derived peptide isolated from the stomach of rats. Since then, evidence for this peptide has been presented for a number of vertebrate species including birds (Figure 3). The obestatin peptide is delimited by two basic amino acid residues (lysines, K) that serve as proteolytic recognition sites in the C-terminal peptide portion of the proghrelin precursor. Avian obestatin displays one notable difference compared to its mammalian counterparts. All mammalian obestatin peptides contain a conserved C-terminal glycine residue (G) that is utilized for amidation of the peptide at this end [32]. Amidation of the C-terminus was thought to be essential for the binding of obestatin to its cognate receptor, originally proposed to be GPR39 [32]. However, all known avian obestatin peptides have a glutamic acid residue (E) in this position (Figure 3). Therefore, it must be assumed that avian obestatin (if it exists) would be 24 amino acids in length and not amidated at its C-terminal end (Figure 4). Zhang et al. [32] also identified an N-terminally truncated 13 amino acid amidated peptide (obestatin 11–23). This peptide fragment was produced by proteolytic cleavage of the full length obestatin peptide at an internal lysine residue (Lys^10^). The same scenario is possible for avian obestatin peptides which contain a conserved lysine at the identical internal position (Figure 3). Thus, a 14 amino acid truncated obestatin peptide (obestatin 11–24) is predicted in birds which would not be C-terminally amidated (Figure 4). To date, there have been no reports of the detection or isolation of obestatin or the truncated peptide in any avian species. Given the low level of homology (46%) between avian and human obestatin peptides (Table 1), it is likely that new avian-specific antibodies will be required to detect the presence of obestatin peptides in blood and tissue samples obtained from birds. It is, however, important to isolate and purify the avian peptide and its fragment to confirm the existence of the predicted differences in structure compared to its mammalian counterpart.

### 3.4. Other Precursor-Derived Peptides?

Another proghrelin-derived peptide that has been detected in circulation in mammals is C-ghrelin which is the C-terminal 66 amino acid peptide containing obestatin that remains after the cleavage of mature ghrelin [13]. The stomach is thought to be the major source of circulating C-ghrelin [13]. To date there is no evidence that this peptide is present in tissue or blood of any avian species, although if it does exist it is predicted to be 65 amino acids in length (Figure 4).

## 4. Proghrelin-Derived Peptide Receptors

Following the initial discovery of rat and human ghrelin peptides as endogenous ligands for the GHS-R, a G protein-coupled 7 transmembrane domain (TMD) receptor [1], Kaiya et al. [2] reported similar findings for chicken ghrelin which was purified based on its ability to bind to the rat GHS-R1a receptor expressed in a mammalian cell line. Subsequently, avian GHS-R genes were identified and their sequence, structure, genomic organization, and expression were studied in chickens [3–5, 8, 19, 33–36]. The findings of Zhang et al. [32] that the GHS-R related orphan receptor GPR39 was thought to be the putative obestatin receptor led to the cloning and characterization of chicken and Japanese quail GPR39 gene orthologs [37, 38]. In light of the subsequent failure to confirm the initial findings of Zhang et al. [32], interest in avian GPR39 genes has declined. However, a brief discussion of GPR39 is included below since this receptor is structurally related to GHS-R and so that meaningful comparisons can be made in the structure and expression of the avian and mammalian genes.

### 4.1. Growth Hormone Secretagogue Receptor (GHS-R)

**Figure 5:**
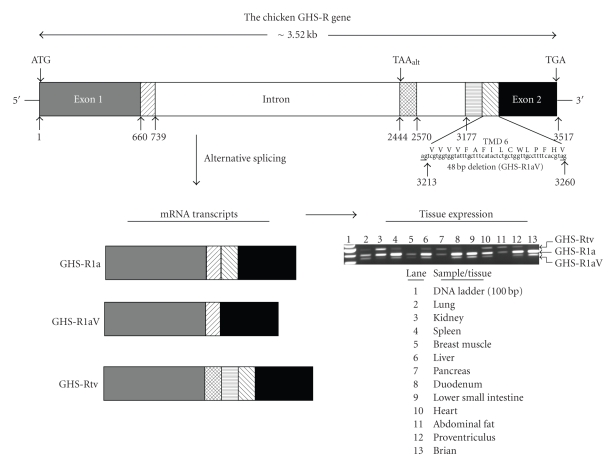
Genomic structure, organization, and expression of the chicken GHS-R gene consisting of two exons separated by a single intron. Different alternative splicing mechanisms are used to produce three transcript variants (GHS-R1a, GHS-R1aV, and GHS-Rtv) that encode proteins of 347, 331, and 220 amino acids, respectively. Sequence encoding a major portion of transmembrane domain 6 (TMD 6), located at the 5′-end of exon 2, is shown and is spliced out of the GHS-R1aV transcript. The GHS-Rtv variant is formed by splicing two portions of intron sequence, one of which contains an alternative stop codon (TAA_alt_) which is predicted to produce a receptor protein truncated after TMD 5. Expression of each of the GHS-R mRNA transcript variants in different tissues obtained from 3-week-old broiler chickens is also shown.

Like its mammalian counterparts, the chicken GHS-R gene, located on chromosome 9, consists of two exons separated by a single intron (Figure 5). The gene encodes a 347 amino acid protein containing 7 TMDs that demonstrates high homology (>70% amino acid identity) with the human protein (Table 1). The first exon contains coding sequence for the first 5 TMDs and the sixth and seventh domains are encoded in exon 2 [33, 34]. Transcription and alternative splicing of the chicken GHS-R gene have been reported to produce three distinct transcripts, GHS-R1a, GHS-R1aV (also referred to as GHS-R1c), and GHS-Rtv (Figure 5). The GHS-R1a transcript is produced by splicing exons 1 and 2 and encodes the full length (347 amino acids) receptor protein with 7 TMDs. The GHS-R1aV transcript variant exhibits a 48 bp deletion at the 5′-end of exon 2 caused by the use of an alternative splice acceptor site during processing of the mRNA transcript [34]. This deleted sequence results in the loss of 16 amino acids from TMD 6. Therefore, the protein produced from this transcript would be predicted to lack a functional sixth domain but contain an intact seventh TMD. Because of this, the GHS-R1aV transcript is thought to encode a nonfunctional receptor similar to the GHS-R1b transcript produced by mammalian GHS-R genes [39]. However, the processed chicken GHS-1aV transcript is not identical to the mammalian GHS-R1b which is generated by termination of transcription within the intron at an alternate stop codon which produces a receptor protein that is truncated after TMD 5. The avian GHS-Rtv transcript has some sequence captured from the intervening intron including an alternate stop codon (Figure 5). This transcript would be predicted to produce a receptor protein that is truncated after TMD 5 and is thus more like the mammalian GHS-R1b [35]. Recently, Japanese quail GHS-R gene transcripts (including a GHS-R1b variant) have been identified that result from additional splicing mechanisms affecting the encoded protein (e.g., N-terminal truncation, loss of TMDs) indicating the potential for new and more complex processing of avian GHS-R mRNA transcripts (GenBank accession nos. AB469019–AB469022). Fang et al. [36] reported a number of sequence polymorphisms present in the chicken GHS-R gene. These occurred in both exons as well as the intron and were, in some cases, related to growth and carcass traits.

Expression of chicken GHS-R1a and GHS-R 1aV transcripts has been detected in all tissues examined with the highest levels of expression of GHS-R1a found in pituitary and hypothalamus [2–5, 8, 18, 33, 34]. The latter finding is consistent with the proposed role of GHS-R in mediating the effects of ghrelin on GH release and food intake. While the GHS-Rtv transcript has only been identified in ovarian cells [35], reexamination of our original findings [8] indicates that it is coexpressed along with the other two transcripts in different tissues in the broiler chicken (Figure 5). In general, the GHS-R1a transcript is the most highly expressed followed by the GHS-R1aV and GHS-Rtv. To date there have been no reports concerning the detection of GHS-R proteins so it is not yet possible to determine if each of the three mRNA transcripts results in an expressed protein in birds. However, the fact that Kaiya et al. [2] were able to utilize the rodent GHS-R1a to screen for chicken ghrelin strongly suggests that the chicken GHS-R1a protein should function in a similar way to its mammalian counterparts. While there has been no systematic attempt to determine the expression of individual GHS-R isoform mRNA, there is preliminary evidence to suggest that there may be differential tissue-specific expression of these receptor variants (Figure 5) and that could have some functional significance.

Very little is currently known about the regulation of the GHS-R gene in birds. Geelissen et al. [33] reported that chicken ghrelin, GH, and corticosterone administered to pituitary explants in vitro down-regulated the production of the GHS-R1a mRNA while thyrotropin-releasing hormone (TRH) was without effect despite its recognized ability to act as a potent GH-releasing factor in chickens [5]. These preliminary results seem to suggest the possibility for ligand-mediated negative feed-back regulation of this gene in birds [33].

### 4.2. GPR39 and the Search for the Obestatin Receptor

Two reports have identified and characterized GPR39 gene orthologs in the chicken and Japanese quail [37, 38]. The chicken GPR39 gene, located on chromosome 7, consists of two exons divided by a single intron and encodes a 462 amino acid 7 TMD protein that exhibits 61% amino acid identity with the human protein (Table 1). The GPR39 gene in birds exhibits very similar genomic organization to the GHS-R gene especially with respect to the positioning of the intron and the inclusion of coding sequence for the sixth and seventh TMDs in the second exon [37]. Within the deduced protein, two cytoplasmic regions located between TMDs 1&2 and TMDs 3&4, and an extracellular loop region (putative ligand-binding region) located between TMDs 6&7 are all highly conserved compared to the human protein suggestive of a similar function for this orphan receptor in birds [37]. The GPR39 gene is widely expressed in different tissues in the chicken and Japanese quail including the gastrointestinal tract (with the highest levels found in duodenum), reproductive tissues, liver, and kidney [37, 38]. Very low levels of expression were observed in the brain and pituitary unlike the GHS-R. Expression in the duodenum was found to increase dramatically during the early posthatch period in chickens, suggesting a potential role in intestinal function and/or maturation [37]. Other than cloning and sequencing GPR39 gene orthologs and determining tissue mRNA expression levels in two avian species, there has been no effort to further characterize any ligand-binding or functional aspects of this GHS-R-related receptor in birds nor has there been any attempt to determine its endogenous ligand.

## 5. Physiological Actions of Proghrelin-Derived Peptides

The focus of this section will be on active ghrelin (the acylated peptide), since there are very few published reports addressing the physiological actions for any other proghrelin-derived peptide in birds. With respect to ghrelin, some of its reported functions are similar to mammals such as its stimulatory effects on GH release and gastrointestinal tract contractility [3–5]. However, there are notable differences such as the effects of ghrelin on food intake and energy homeostasis. The previous sections have clearly demonstrated that both ligand and receptor are highly conserved between mammalian and avian species. This is reflected in the finding by Kaiya et al. [2] that chicken acylated-ghrelin was capable of binding to and signaling through the rat GHS-R in cultured cells. Therefore, structural variations most likely do not account for observed differences in function. In the following sections what is currently known about the pleiotropic actions of ghrelin in birds will be discussed in relation to what has been reported in mammals.

### 5.1. Hormone Releasing Activity

One of the principal effects of ghrelin in mammals is to stimulate GH release from the pituitary, and its ability to bind to the GHS-R formed the basis for its initial discovery [1, 40]. In birds, administration of ghrelin (human, rat, or purified chicken peptide) by intraveneous (iv) injection is known to transiently elevate circulating GH levels in both young and adult chickens in a dose- and time-dependent manner [2, 41, 42]. While it is clear that acylated-ghrelin working through the GHS-R promotes GH release in chickens, the exact mechanism(s) involved has not been completely defined in these studies. It was shown that both rat and synthetic chicken ghrelin peptides bound to and activated the GHS-R in vitro (cell culture assay system) with equal potency and stimulated GH release into plasma in vivo with similar dose-response curves [2]. Using dispersed pituitary cells, Baudet and Harvey [42] showed that human ghrelin induced a dose-dependent GH release with potency comparable to GHRH indicating that ghrelin was capable of acting directly on pituitary somatotrophs. These findings are supported by the high level of GHS-R1a mRNA expression found in the chicken pituitary [33, 34]. However, this does not rule out the possible role of the hypothalamus in mediating the effects of ghrelin on GH release in vivo. Ahmed and Harvey [41] detected ghrelin protein expression in specific regions of the chicken hypothalamus. They suggested ghrelin, expressed locally in the central nervous system (CNS), might also participate in regulation of the hypothalamic-pituitary-GH axis. In mammals, it has also been suggested that ghrelin acts peripherally to effect GH release via stimulation of vagal afferent nerves [40]. This possibility has not yet been explored in birds.

The release of corticosterone from the adrenal gland into circulation represents a concurrent hormone releasing activity affected when ghrelin is administered to birds [2, 17]. In fact, it has been observed that the effect of ghrelin on corticosterone release is more pronounced in chickens as compared to other species including rats and humans [17]. Kaiya et al. [2] reported that iv injections of human or chicken ghrelin into young growing chicks resulted in increases in plasma corticosterone in a dose- and time-dependent manner and suggested that ghrelin could be an important regulator of adrenal function in birds. Saito et al. [17] found that icv injection of chicken ghrelin also increased plasma corticosterone in a dose- and time-dependent manner in neonatal chicks. Moreover, coadministration of the CRF receptor antagonist astressin reduced the effect of ghrelin on plasma corticosterone. Together, these findings suggest a mechanism of action for ghrelin in regulating the hypothalamic-pituitary-adrenal (HPA) axis via CRF and adrenocorticotropic hormone (ACTH) [17]. These findings are supported by expression of GHS-R1a mRNA in both the hypothalamus and the adrenal gland consistent with the potential for both local and CNS actions by ghrelin on the HPA [2, 33].

### 5.2. Food and Water Intake

The single most significant difference in ghrelin function between birds and mammals concerns its role in the regulation of food intake. Ghrelin is a potent orexigen in mammals when injected centrally or peripherally [1, 13, 40]. Numerous studies with birds have now clearly shown that ghrelin administered icv inhibits food intake in a dose-dependent manner under both fed and fasted conditions [2–5, 17, 29, 43, 44]. In contrast, central administration of des-acyl ghrelin had no effect on food intake in neonatal chicks [45]. However, peripheral administration of ghrelin produced some conflicting findings with respect to its effects on food intake [18, 29, 46]. These discrepancies do not appear to involve unique (to birds) differences in the structure of ghrelin and the GHS-R since icv injection of synthetic heterologous ghrelins (rat and bullfrog) produced comparable anorexigenic effects to those observed with homologous (chicken) ghrelin [44].

In mammals, the orexigenic effect of ghrelin is thought to involve the activation of hypothalamic neurons expressing neuropeptide Y (NPY) and agouti-related peptide (AgRP) as well as downstream neurons expressing other orexigenic peptides such as orexin [40]. Thus, a logical explanation for the different effect of ghrelin on food intake in birds would involve a different site(s) and/or mechanism(s) of action for ghrelin in the CNS. Saito et al. [17] clearly showed that ghrelin administered icv to neonatal chicks failed to increase hypothalamic NPY mRNA expression. Moreover, this group also found that NPY-induced feeding could be antagonized by coadministration of ghrelin suggesting the involvement of separate neuronal/neuroendocrine pathways. They concluded that central administration of ghrelin to birds does not activate NPY-expressing neurons, but instead works via CRF and the HPA axis to bring about anorexia and increased circulating corticosterone levels. The CRF receptor antagonist astressin was able to attenuate ghrelin-induced reduction in food intake [17]. Khan et al. [46] recently reported that coadministration of the nitric oxide synthase (NOS) inhibitor N^G^-nitro-L-arginine methylester (L-NAME) to neonatal chicks attenuated the anorexigenic effect of CRF suggesting a role for NO in mediating the central effects of CRF. Interestingly, this group found no effect of coadministration of L-NAME on ghrelin-induced anorexia which implies that a CRF/NO axis may not mediate ghrelin's actions on food intake in birds.

Administration of ghrelin to mammals produces an orexigenic response whether administered centrally or peripherally [40]. In birds, the effects of ghrelin administered peripherally produced conflicting results ranging from having no effect [18] to stimulating [29] or inhibiting [47] food intake in chickens and Japanese quail. It has been suggested that very high peripheral doses of ghrelin could bypass the blood-brain barrier and act directly on the hypothalamus, whereas lower doses might work via stimulation of vagal afferent nerves in the periphery that influence central feeding regulatory circuits via neural relays in the nucleus of the solitary tract (NST) of the brain stem [5]. In support of the latter mechanism, vagotomy in mammals is known to inhibit ghrelin's ability to stimulate GH release and food intake [40]. However, this has not been studied in birds. In general, it is likely that different neurocircuits and neuroendocrine factors mediate ghrelin-induced changes in feeding behavior in birds as compared to mammals. Also important are the sites of GHS-R expression, as these will determine which neuronal circuits are impacted by ghrelin. Further study is required to more fully elucidate the nature and function of central feeding circuits and the neuroendocrine factors involved in birds.

It has been reported that centrally administered ghrelin acts as an anti-dipsogenic peptide in chickens [48]. Ghrelin-induced inhibition of water intake occurred under *ad libitum* and water-deprived conditions. In contrast, des-acyl ghrelin had no effect on water intake suggesting a role for GHS-R in mediating this effect. Recently, obestatin (synthetic chicken peptide) was reported to decrease water consumption in a line of chickens selected for low body weight [49]. In general, the mechanisms mediating the effects of proghrelin-derived peptides on water consumption remain unknown.

### 5.3. Energy Homeostasis

Geelissen et al. [47] reported that peripheral (iv) administration of ghrelin to week-old male broiler chickens reduced the respiratory quotient (RQ) without affecting metabolic rate or heat production. A decrease in RQ could indicate that ghrelin induced a decline in lipogenesis and increased fatty acid oxidation in peripheral tissues and such effects would signal a switch in fuel utilization from carbohydrate to lipid. However, Geelissen et al. [47] observed no changes in plasma levels of glucose, triglycerides, fatty acids, or T_3_ in response to ghrelin administration. In contrast, Shousha et al. [29] reported that both peripheral (ip) and central (icv) administration of ghrelin (rat peptide) to adult male Japanese quail invoked transient dose-dependent increases in body temperature indicative of increased thermogenesis and energy expenditure. Most of these observations in birds are in direct opposition to the reported effects of ghrelin in mammalian species in which it is reported to stimulate appetite, reduce fat oxidation (increase RQ), and induce weight gain without affecting heat production [13, 40]. Recently, Buyse et al. [50] reported that a single iv injection of chicken ghrelin into *ad libitum* fed day-old male broiler chicks reduced mRNA expression of fatty acid synthase (FAS) and two lipogenic-related transcription factors (SREBP-1 and PPAR-*γ*) in liver. These findings indicated a ghrelin-induced reduction in fatty acid biosynthesis at the main tissue site for lipogenesis in birds. Interestingly, the effect of peripheral ghrelin administration on expression of these same lipogenic genes in the diencephalon was opposite to that observed in liver. It was suggested that the anorexic effect of ghrelin in birds might involve regulation of FAS expression in hypothalamic neurons [50]. Again, these findings contrast sharply with what has been observed in mammals suggesting an opposite action of ghrelin on energy homeostasis which coincides with its opposite effect on food intake in birds.

### 5.4. Reproduction

In mammals, ghrelin and GHS-R are expressed in gonadal tissue as well as in hypothalamic neurons that function in the gonadotropic axis indicative of a regulatory role in reproduction [13]. Similar observations have been made in birds. Yoshimura et al. [51] detected ghrelin mRNA and protein expression in mucosal epithelial cells of the infundibulum and magnum portions of the oviduct in laying Japanese quail. Recently, this same group identified ghrelin in different components (i.e., yolk, albumin) of fresh fertilized chicken eggs with higher levels detected in yolk as compared to albumin [52]. While ghrelin concentration of total egg contents (i.e., yolk, albumin, and embryo) did not change during the first 5 days of incubation, it was suggested that it could play a role in embryonic cells during early phases of development of chicken embryos. A possible early developmental role for ghrelin (perhaps initially derived from maternal sources) in birds is further supported by the findings of Gahr et al. [53] who detected GHS-R expression in early developing chicken embryos and observed a 2.5-fold increase between days 4 and 5 of incubation.

There have several reports on the expression of ghrelin and GHS-R in ovarian tissue and cells suggesting the potential for autocine/paracrine effects of the proghrelin system in the avian ovary [35, 54, 55]. Administration of ghrelin or a ghrelin analog (ghrelin 1–18) to ovarian granulosa cells in culture induced markers of proliferation while decreasing markers of apoptosis, as well as stimulating the release of progesterone, estradiol, arginine-vasotocin (AVT), and insulin-like growth factor-I (IGF-I) in cultured ovarian follicular tissue fragments [35]. This suggested a role for ghrelin in regulating key ovarian functions (i.e., apoptosis, cellular proliferation, and steroid and peptide hormone secretion) in chickens mediated by GHS-R1a and postreceptor signal transduction mechanisms involving tyrosine kinase (TK), mitogen-activated protein kinase (MAPK), cyclin-dependent kinase (CDK), and protein kinase A (PKA) [54, 55].

### 5.5. Gastrointestinal Function

Ghrelin affects various aspects of gastrointestinal function including exocrine secretion, epithelial cell viability, and GI tract motility in mammals [13, 40]. Ghrelin immuno-reactivity has been detected predominantly in the mucosal layer of the proventriculus, duodenum, jejunum, ileum, caecum, and colon but not in the myenteric plexus of chickens and African ostrich chicks [20, 21, 56–58]. In general, the greatest number of ghrelin-immunopositive cells is found in the proventriculus and the number of these cells tends to increase with age. The high level of ghrelin mRNA and protein expression in proventriculus is consistent with a possible endocrine role for this organ in birds. A similar endocrine role has been proposed for the proventriculus in regulating circulating levels of leptin and proglucagon-derived peptide hormones [59]. In mammals, it has been reported that the stomach accounts for the majority of proghrelin-derived peptides in circulation [13, 40].

Studies utilizing isolated gut sections in culture from young growing chickens found that ghrelin stimulated contraction of the upper (crop and esophagus) and lower (colon) portions of the GI tract but had weaker effects on the intervening (proventriculus, duodenum, jejunum) regions which were more sensitive to the ghrelin-related peptide motilin [31]. Chicken ghrelin was found to have region-specific effects on contraction, whereas human and rat ghrelin produced only weak responses. Also, des-acyl ghrelin did not affect motility suggesting that a localized effect of ghrelin on GI tract motility was mediated through GHS-R which is differentially expressed in the crop (smooth muscle layer) versus the proventiculus (smooth muscle layer and enteric neurons) in chickens [31]. In a related study, Khan et al. [60] reported that icv administration of growth hormone releasing peptide-6 (GHRP-6, a ghrelin mimetic) inhibited food intake transiently but did not affect retention of food in the crop, proventriculus, or gizzard in neonatal chicks. Together, these findings suggest localized as opposed to central effects of ghrelin on gut motility in birds. However, it is not known if these effects on gut contractility have any influence on the regulation of food intake and nutrient utilization [4].

### 5.6. Behavioral Effects

As part of the studies of the various physiological actions of ghrelin in birds, it has been noted that this peptide also has important impacts on behavior. For example, ghrelin has been reported to induce sleep-like behavior in a dose-dependent manner when administered centrally to fed or fasted neonatal chicks suggesting that this behavior may be related to its inhibitory effect on food intake [45, 61]. However, the sleep-like behavior was preceded by a short period (less than 30 minutes) of hyperactivity and increased vocalization [61]. Since CRF also induces hyperactivity when administered icv, this behavior could have resulted by the actions of ghrelin on CRF expression in the CNS [5]. Widespread expression of ghrelin and GHS-R in different regions of the brain beyond the feeding centers located within the hypothalamus is consistent with direct involvement of the ghrelin system in controlling behavior [17, 33]. Recently, Carvajal et al. [62] reported that central administration of ghrelin induced fearful and/or anxious behavior and impaired memory retention in a dose-dependent manner in addition to inducing anorexia in neonatal chicks. The interrelationship of behavior and metabolism is an important new area to explore since it is now recognized that these processes are regulated by shared neuronal circuits in the hypothalamus and that sleep and arousal may actually represent mechanisms involved in energy conservation and expenditure, respectively [63].

## 6. Conclusions and Future Directions

This review discussed what is currently known about the proghrelin system in birds. While the genes encoding preproghrelin and GHS-R and their protein products are reasonably conserved in avian species, there are some significant differences in the function of the proghrelin system in birds as compared to mammals. The opposite effect of ghrelin on food intake undoubtedly involves the actions of different neural circuits and neuroendocrine pathways downstream of the ligand-receptor binding. Perhaps, by studying ghrelin's effects on food intake, it will be possible to learn more about the specific mechanisms governing feeding behavior in birds and how that regulation differs from what is known for other vertebrate species.

Despite a growing body of evidence for a proghrelin system in birds, a number of unanswered questions remain. Is the obestatin peptide hormone actually produced? If so, what are its structural characteristics and functions? Is it amidated at its C-terminal end? What, if any, are the physiological actions of des-acyl ghrelin? Are there other peptides or fragments produced from the proghrelin precursor? What proghrelin-derived peptides circulate in the blood and in what proportions? Are there additional GHS-R isoforms or related-receptors expressed and how do they mediate the actions of proghrelin-derived peptides? Why are some actions of ghrelin conserved (e.g., growth hormone release) while others are markedly different (e.g., food intake)? Working to answer these and other relevant questions about the avian proghrelin system presents a unique opportunity to better understand the structure and function of this important endocrine system and how it has evolved in different animal species including humans.

## Figures and Tables

**Table 1:** Characteristics of chicken proghrelin system components.

Component^1^	Size (aa)	Chromosome	Homology (%)^2^
(1) Preproghrelin	116	12	36
(i) Signal Peptide	23		30
(ii) Ghrelin	26/28^3^		54
(iii) C-Terminal Peptide	65		31
(iv) Obestatin	24^4^		46
(2) Growth Hormone Secretagogue Receptor (GHS-R)		9	
(i) GHS-R1a	347		76
(ii) GHS-R1aV	331		76
(iii) GHS-Rtv	220		78
(3) GPR39^5^	462	7	61

^1^Information for size and homology derived from: GenBank accession nos. NP_001001131, NP_989725, and NP_001073574 for preproghrelin, growth hormone secretagogue receptor (GHS-R), and GPR39, respectively.

^2^Based on amino acid identities compared to corresponding human sequence (NP_001128413, NP_004113, and NP_001499 for preproghrelin, growth hormone secretagogue receptor (GHS-R), and GPR39, resp.).

^3^The chicken ghrelin peptide contains two arginine residues (RR) at its C-terminal end that serve as a processing signal for proteolytic cleavage and are removed by the action of carboxypeptidase to give rise to the mature 26 amino acid peptide [2]. The human ghrelin peptide contains a proline and arginine pair (PR) at its C-terminal end and gives rise to a 28 amino acid mature peptide because these two amino acids are retained.

^4^The human obestatin peptide (23 amino acids) contains a glycine residue at its C-terminal end that is used for amidation. In contrast, chicken obestatin (24 amino acids) contains a glutamic acid residue at this site and is therefore one amino acid longer than the human peptide and is most likely not amidated.

^5^Information for GPR39 is included because it is a G protein-coupled receptor related to GHS-R. However, it is now generally accepted that this orphan receptor is not the putative obestatin receptor in mammals and this is assumed to apply to birds as well.
